# Carbon-rich materials with three-dimensional ordering at the angstrom level

**DOI:** 10.1039/d0sc02422h

**Published:** 2020-06-01

**Authors:** Shixin Fa, Masanori Yamamoto, Hirotomo Nishihara, Ryota Sakamoto, Kazuhide Kamiya, Yuta Nishina, Tomoki Ogoshi

**Affiliations:** a Department of Synthetic Chemistry and Biological Chemistry , Graduate School of Engineering , Kyoto University , Katsura, Nishikyo-ku , Kyoto , 615-8510 , Japan . Email: ogoshi@sbchem.kyoto-u.ac.jp; b Institute of Multidisciplinary Research for Advanced Materials , Tohoku University , 2-1-1 Katahira, Aoba-ku , Sendai , Miyagi 980-8577 , Japan; c Advanced Institute for Materials Research (WPI-AIMR) , Tohoku University , Katahira 2-1-1, Aoba-ku , Sendai , Miyagi 980-8577 , Japan; d Department of Energy and Hydrocarbon Chemistry , Graduate School of Engineering , Kyoto University , Katsura, Nishikyo-ku , Kyoto , 615-8510 , Japan; e Graduate School of Engineering Science , Osaka University , 1-3 Machikaneyama , Toyonaka , Osaka 560-8531 , Japan; f Research Center for Solar Energy Chemistry , Osaka University , 1-3 Machikaneyama , Toyonaka , Osaka 560-8531 , Japan; g Research Core for Interdisciplinary Sciences , Okayama University , 3-1-1 Tsushima-Naka, Kita-ku , Okayama , 700-8530 , Japan; h WPI Nano Life Science Institute (WPI-NanoLSI) , Kanazawa University , Kakuma-machi , Kanazawa , Ishikawa 920-1192 , Japan

## Abstract

Carbon-rich materials, which contain over 90% carbon, have been mainly synthesized by the carbonization of organic compounds. However, in many cases, their original molecular and ordered structures are decomposed by the carbonization process, which results in a failure to retain their original three-dimensional (3D) ordering at the angstrom level. Recently, we successfully produced carbon-rich materials that are able to retain their 3D ordering at the angstrom level even after the calcination of organic porous pillar[6]arene supramolecular assemblies and cyclic porphyrin dimer assemblies. Other new pathways to prepare carbon-rich materials with 3D ordering at the angstrom level are the controlled polymerization of designed monomers and redox reaction of graph. Electrocatalytic application using these materials is described.

## Abstract 2

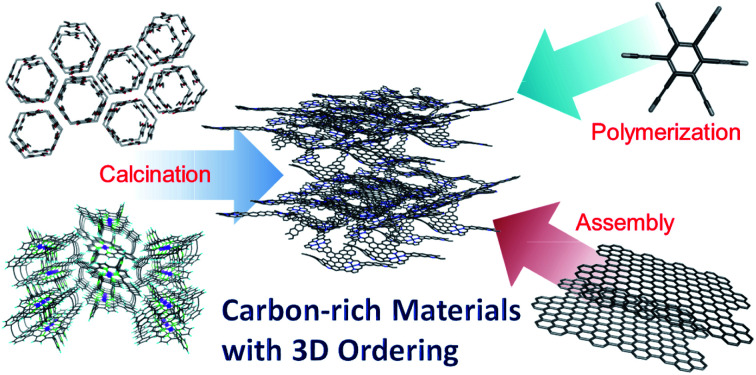
We discuss the preparation and application of various carbon-rich materials with three-dimensional ordering at the angstrom level.

## 1. Introduction

**Fig. 1:**
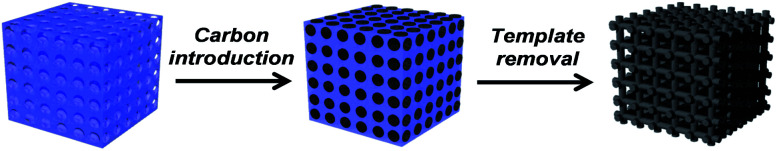
Template technique to prepare carbon-rich materials with 3D ordering.

Carbon-rich materials with composition ratios of >90% have been investigated widely because of their robustness against mechanical, chemical, heat, and abrasion stimuli, and have various applications, such as adsorbents, catalyst supports, and electrode materials.^1^–^10^ Such carbon-rich materials have been fabricated chiefly by the carbonization of organic compounds and polymers. However, in most cases, their original molecular and ordered structures are ruined during the carbonization process, which results in the loss of their original three-dimensional (3D) ordering. Since the late 1990s, a series of efforts to realize carbon-rich materials with 3D ordering derived from the starting materials (*i.e.*, porosity and crystallinity) has been made. A distinctive approach includes a template technique (Fig. 1):^1^–^7^ First, template materials are covered or impregnated with carbon sources to form nanocomposites. Second, the hard templates are removed, for example, by chemical etching, to liberate the carbon frameworks. As a result, nanostructured carbon-rich materials are obtained as negative replicas of the hard templates. The template carbonization is a straightforward method to obtain carbon-rich materials with 3D ordering; however, there is a limitation in the scale of the ordered structures with pores that can be controlled at the molecular level. To avoid this limitation, the calcination of a series of molecule-based porous frameworks with controlled pore sizes and 3D ordering structures at the molecular scale has been investigated,^8^–^10^ such as porous coordination polymers (PCPs) or metal–organic frameworks (MOFs), and covalent organic frameworks (COFs). However, their carbonization tends to damage the original 3D structures, so the fine carbon architectures at the angstrom level are lost. Recently, we have discovered that the carbonization of organic porous supramolecular assemblies constructed from pillar[6]arenes, which are hexagonal prism-shaped macrocyclic compounds, leads to carbon-rich materials with micropores controlled precisely at the angstrom level.^11^ We have also discovered that calcination of cyclic porphyrin dimer assemblies yields carbon-rich materials with regular crystalline structures at the angstrom level.^12^ Furthermore, we have synthesized graphdiynes, which are carbon-rich materials with regular hexagonal structures at the angstrom level, by the interfacial polymerization of designed monomers.^13^,^14^ A controlled electrochemical redox reaction of graphene is another way to produce carbon-rich materials with controlled interlayer distances at the angstrom level.^15^ This series of studies provides a new method to synthesize porous carbon materials containing controlled pores and structures at the angstrom level. From the viewpoint of application, carbon-rich materials with 3D ordering have attracted increasing attention as electrocatalysts, owing to their high surface area, high electrical conductivity, and designed structure at the molecular scale. Unique electrocatalytic activities resulting from their 3D ordering structures are also described.

## 2. Porous carbons: calcination of pillar[6]arene assemblies

Preparation of porous carbons from organic molecules is limited because of the high demand of thermo-stability for high-temperature carbonization. Such molecules, which include those from simple aromatic monomers (*e.g.*, benzene,^16^,^17^ thiophene,^18^,^19^ and phenyltrimethylsilane^20^) to more complex ones (*e.g.*, triptycene^21^ and porphyrin derivatives^22^), have been used as carbon sources to prepare porous carbons. Typically, these monomers are preliminarily polymerized before carbonization. This step is necessary to avoid decomposition or fusion of the molecules during the pyrolysis treatment and increases the porosity and physiochemical stability. The subsequent treatment of the polymers at high temperature under inert gases produces porous carbon materials, which possess a high surface area and pore volume. However, because of the mediocre controllability of the pore size and shape during polymerization, ideal porous carbon materials with well-defined pores at the sub-nanoscale are unprocurable using this method.

**Fig. 2:**
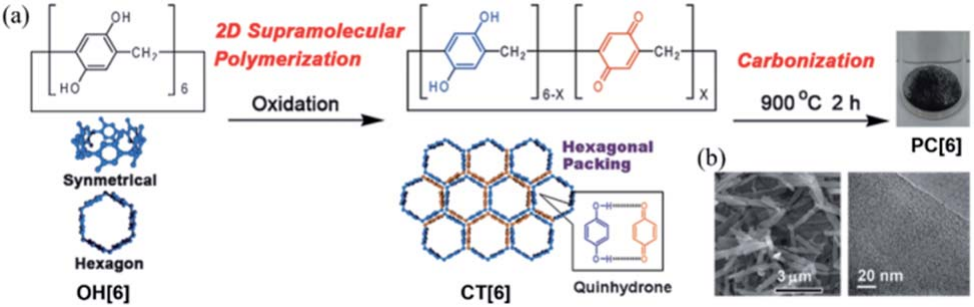
(a) 2D supramolecular polymerization by oxidation of **OH[6]**, and porous carbon (**PC[6]**) prepared by carbonization of **CT[6]**. (b) SEM and TEM images of **PC[6]**. Reproduced with permission from ref. 11. Copyright 2015 Wiley-VCH Verlag GmbH & Co. KGaA.

Using macrocyclic molecules as starting compounds is a good solution to obtain porous carbons with control at the angstrom level. If the macrocyclic structure is retained after carbonization, porous carbon with angstrom-scale pores that result from the cavity size of the macrocyclic compounds can be obtained. Our group has reported the fabrication of porous carbon **PC[6]** with well-defined angstrom-scale pore sizes (Fig. 2).^11^ We used a symmetric hexagonal macrocyclic compound, pillar[6]arene, as a starting compound. By oxidation of the precursor pillar[6]arene **OH[6]**, which consists of six hydroquinone units, in a homogeneous solution using an oxidant, two-dimensional (2D) hexagonal close-packed assembly **CT[6]** was precipitated as a result of the multiple inter-molecular charge transfer interaction between hydroquinone and benzoquinone moieties of pillar[6]arenes (Fig. 2a). Owing to the hexagonal structure of pillar[6]arene, **CT[6]** assembled with a hexagonal close-packed structure. In the 2D assembly **CT[6]**, the pore size was determined by using molecular-probe gases and vapor. The pore size of **CT[6]** was 4.04 Å, which was well maintained from the cavity size of **OH[6]** (4.10 Å). The assembled structure of **CT[6]** was a fiber structure. After carbonization at 900 °C under an inert gas atmosphere, porous carbon **PC[6]** was obtained. Fiber structures were retained even after carbonization from scanning electron microscopy (SEM) measurements (Fig. 2b). In the transmission electron microscopy (TEM) image, numerous white dots of a size less than 1 nm were observed, which indicated that the pore sizes of **PC[6]** were uniformly at the angstrom scale (Fig. 2a). By using molecular-probe gases and vapor, the pore size of **PC[6]** was determined to be 4.09 Å, which was very close to that of **OH[6]** and **CT[6]**. This was the first preparation of porous carbon fibers with a pore size that can be precisely controlled at the angstrom level. There is only one example of preparation of porous carbons from pillar[*n*]arenes. However, chemical structures of pillar[*n*]arenes are phenolic groups, which are similar to good carbon sources, phenolic resins. Therefore, pillar[*n*]arenes should be useful carbon sources to produce various porous carbons. Furthermore, pillar[*n*]arenes have high functionality, thus design of porous carbons with functions is next target.

## 3. Crystalline carbons: calcination of porphyrin dimers

**Fig. 3:**
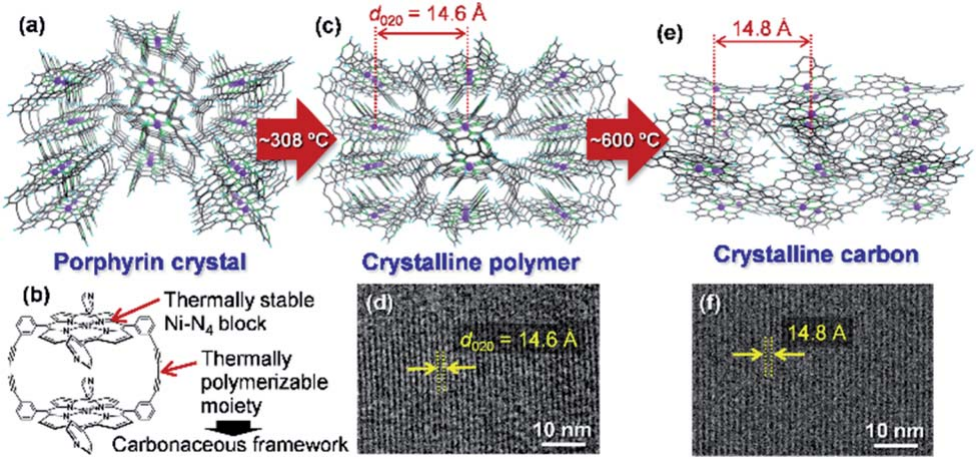
(a) Crystal structure of Ni_2_-CPD_Py_. (b) Structure of a Ni_2_-CPD_Py_ molecule. (c) Crystal structure of polymer formed by thermal treatment. (d) TEM image of the polymer. (e) Expected atomic-level structure of crystalline carbon. (f) TEM image of crystalline carbon. Reproduced with permission from ref. 12. Copyright 2017 Nature Publishing Group.

Carbon-rich materials except graphite generally consist of non-crystalline matrices that exhibit disordered and amorphous structures, thus it has been a challenging target to produce carbon-rich materials with the original crystal structures by the calcination process. In 2017, the direct conversion of organic crystals into structurally defined carbon-rich materials was achieved by using a supramolecular assembly of Ni-containing cyclic porphyrin dimers (Ni_2_-CPD_Py_)^23^ as a precursor (Fig. 3).^12^

The molecular crystal (Fig. 3a) consists of Ni_2_-CPD_Py_ molecules (Fig. 3b) that possess thermally stable Ni–N_4_ blocks (porphyrin center) and thermally polymerizable diacetylene moieties. When the Ni_2_-CPD_Py_ crystal is heat treated, the diacetylene moieties are thermally polymerized to form a crystalline polymer (Fig. 3c). Ni, the central porphyrin cation, is regularly arrayed to form the (020) plane (*d*-spacing is 14.6 Å). The TEM image of the polymer clearly shows the (020) plane (Fig. 3d). Upon further heat treatment up to 600 °C, the polymer framework is converted into a carbon-rich material with retention of the ordered structure of the crystalline polymer as well as the Ni–N_4_ structure (Fig. 3e). Since the positions of Ni atoms are almost unchanged, its TEM image (Fig. 3f) is almost the same as that of the crystalline polymer (Fig. 3d). The whole synthesis procedure of such crystalline carbon materials only involves the carbonization of Ni_2_-CPD_Py_ crystals above 600 °C without any complex experimental step. X-ray absorption fine structure analysis revealed that Ni is divalent and the Ni–N_4_ coordination structure is retained. The resulting carbon-rich materials possess ordered frameworks together with molecular-derived functional blocks, which exhibit unique electrocatalysis towards selective CO_2_ reduction. The key factor of this approach is a rational molecular design of precursors, *i.e.*, the combination of thermally stable block (*e.g.*, metal porphyrin) and thermally polymerizable moiety (*e.g.*, diacetylene). Recently, we are exploring other building blocks to achieve a variety of carbon-rich materials with 3D ordering regarding improved porosities, different framework morphologies as well as chemical structures including metal species. For instance, ethynyl group has been found to work as an alternative thermally polymerizable moiety, giving crystalline carbons with developed microporosity.^24^

## 4. Carbons with regular hexagonal structures: graphdiynes

**Fig. 4:**
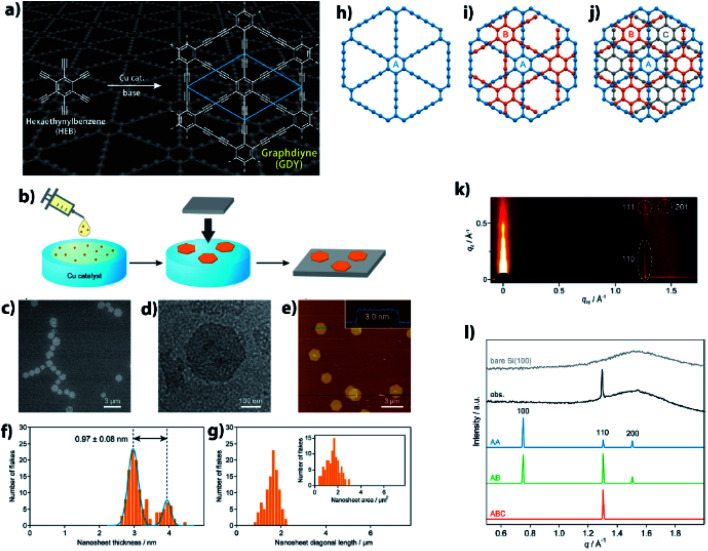
(a) Synthetic scheme and chemical structure of **GDY**. (b) Schematic illustration for the gas/liquid interfacial synthesis. (c) SEM micrograph of **GDY-1** on HMDS/Si(100). (d) TEM micrograph on an elastic carbon grid. (e) AFM topographic image on HMDS/Si(100) and its cross-sectional analysis along the blue line. (f) AFM thickness histogram (orange bars) and its Gaussian fitting (blue lines) (g) AFM domain size (diagonal length) and domain area (inset) histograms. (h–j) AA, AB, and ABC stacking patterns for **GDY**. (k) 2D GIWAXS pattern on Si(100). (l) Experimental and simulated in-plane 2D GIWAXS patterns for the AA, AB, and ABC configurations. An experimental diffraction pattern for bare Si(100) is also shown as a reference. Reproduced with permission from ref. 13. 2017 American Chemical Society.

Precise synthesis of carbon-rich materials with controlled lattice and chemical structures is one of the dreams of organic chemists,^25^,^26^ and this desire was stimulated by the realization of graphene in 2004.^27^ One of the approaches for the precise synthesis involves graphdiyne (**GDY**),^13^,^28^,^29^ which corresponds to a 2D allotrope of carbon. Like graphene, **GDY** features a π-conjugated 2D hexagonal lattice but possesses a different bonding structure in which both sp and sp2 carbons coexist (Fig. 4a). **GDY** is synthesized through multiple alkyne–alkyne dimerizations of an organic monomer, hexaethynylbenzene (HEB). Since the first report on the synthesis of **GDY** by Li and Liu,^30^ a series of synthetic methods has been proposed for **GDY**.^13^Fig. 4b shows one such approach, a gas/liquid interfacial synthesis that was demonstrated by Sakamoto and Nishihara.^31^ The authors employed a gas/liquid interfacial synthesis for the fabrication of functional metal–organic nanosheets relying on a series of coordination bondings,^32^–^35^ which was then applied to **GDY** requiring irreversible carbon–carbon bond formation. Under an Ar atmosphere at room temperature, HEB in dichloromethane and toluene was placed gently onto the surface of an aqueous solution containing Cu(OAc)_2_ and pyridine as the alkyne dimerization catalyst and base, respectively. The organic solvent evaporated spontaneously, and the resultant gas/liquid interface served as a 2D reaction space that facilitated the HEB monomer to form **GDY** with a 2D framework. In addition, the amount of HEB was set low (20 nmol for an aqueous surface of 38 cm^2^) to facilitate thinner **GDY** formation. As a result, the polymerization took place at the gas/liquid interface to generate **GDY** nanosheet domains. A series of microscopy investigations was applied to the **GDY** nanosheet transferred onto flat substrates, which disclosed its regular hexagonal domains reminiscent of its 2D hexagonal lattice (Fig. 4c–e). The thickness and lateral size of the **GDY** hexagon were histogramized, as shown in Fig. 4f and g, from the AFM images, and featured narrow distributions with medians of 2.97 (major) and 3.94 nm (minor) for the thickness, and of 1.51 μm for the lateral domain size. The thickness corresponded to only 7–9 **GDY** layers considering the inter-layer distance (0.34 nm, *vide infra*) and the interaction between the **GDY** domain and AFM tip, and the mean size and area indicated that 2 000 000 HEB molecules coupled together per **GDY** hexagon. One of the major unsolved issues for **GDY** had been the determination of its stacking pattern among the layers. Here, three types of stacking patterns were considered (AA, AB, ABC; Fig. 4h–j), and the **GDY** hexagon was then subjected to synchrotron grazing incidence 2D wide-angle X-ray scattering (2D GIWAXS; Fig. 4k), with its in-plane diffraction profile depicted in Fig. 4l together with those simulated from the three stacking structures. The single experimental diffraction in the measurement range was reproduced solely by the ABC stacking pattern with an assignment of the 110 diffraction. Note that the result was consistent with the selected area electron diffraction associated with TEM, which was applied to a **GDY** sample fabricated by a liquid/liquid interfacial synthesis.^31^ The diagonal 111 and 201 diffractions in 2D GIWAXS (Fig. 4k) allowed the authors to quantify the in-plane and out-of-plane lattice constants as *a* = *b* = 0.96 nm and *c* = 1.02 nm (0.34 nm for the interlayer distance), respectively. This series of results provided evidence for the precise synthesis of **GDY**.

Other synthetic research directions for **GDY** include post-synthetic modifications to implant heteroatoms^25^,^36^,^37^ and the creation of **GDY** analogues by customizing the monomer molecule, HEB.^14^,^38^

## 5. Carbons by assembling graphene materials

**Fig. 5:**
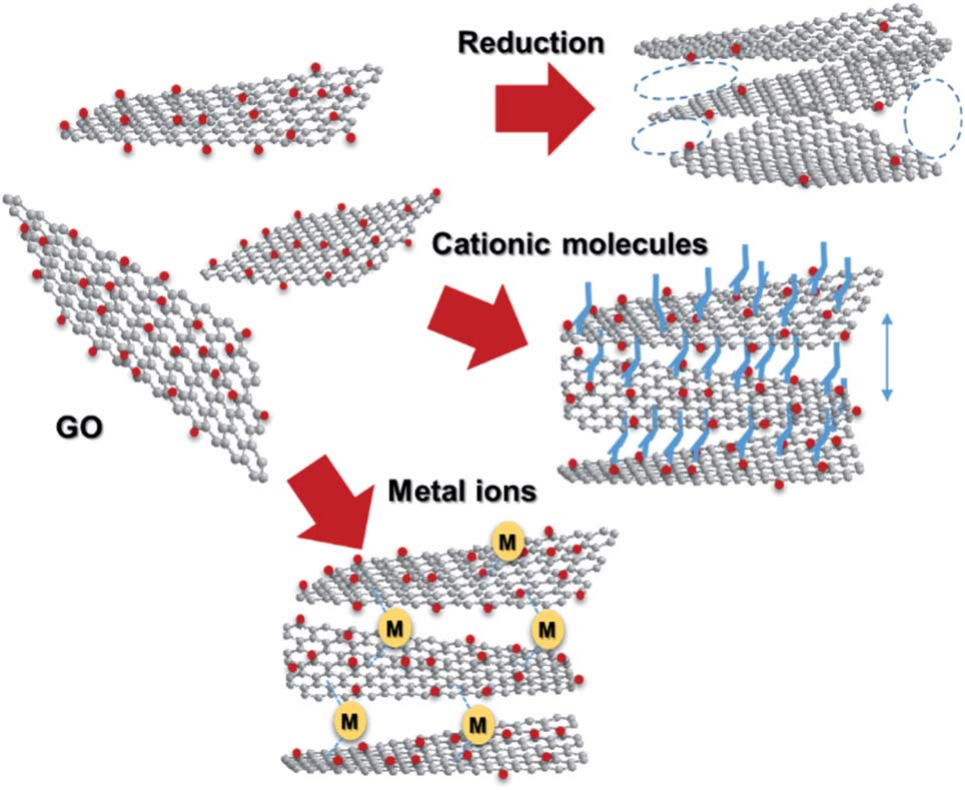
Assembling GO into 3D architectures.

Graphene, a 2D allotrope of carbon, has excellent electron mobility, thermal conductivity, mechanical strength, optical transparency, and specific surface area, and has attracted much attention from researchers in chemistry, physics, materials science, and energy devices. Graphene has the potential for application in high-performance nanocomposites, catalysts, energy storage devices, electronics and optoelectronics, and biological and chemical sensors. However, in some cases, graphene cannot exhibit its inherent performance owing to aggregation or stacking. Processing graphene into a 3D framework may be a solution to the problem. Recently, the fabrication of graphene material has been demonstrated as an effective method to create a free-standing 3D architecture such as hydrogel, aerogel, foam, and sponge. These 3D architectures have low density, high porosity, large surface area, excellent electrical conductivity, and stable mechanical properties. Therefore, assembled graphene material shows potential in many application fields such as supercapacitors, batteries, sensors, catalysts, filters, and absorbents.^39^–^41^ This section introduces how to assemble 2D graphene into a 3D architecture. Graphene oxide (GO), which is easy to synthesize and whose structure can be controlled, is frequently used as a starting material for graphene assembly (Fig. 5). GO has a negative zeta potential and is highly dispersed in water and polar solvents. When the zeta potential becomes imbalanced, the gelation of GO occurs, which forms a GO hydrogel. Using this principle, mixing cationic molecules with GO can control the interlayer distance when GO sheets are stacked. Assembled graphene with an optimum layer distance enables control of the selectivity as a filtration membrane^42^ and catalyst.^43^ The self-assembly of GO sheets includes three steps; (1) reduction with a reducing agent and hydrothermal process, (2) the addition of metal ions, biomolecules and polymers, and (3) lyophilization. In addition, graphene assembly can be produced by adsorbing GO on the surface of spherical polystyrene balls or silica nanoparticles, followed by heat-treatment and removal of the template.

**Fig. 6:**
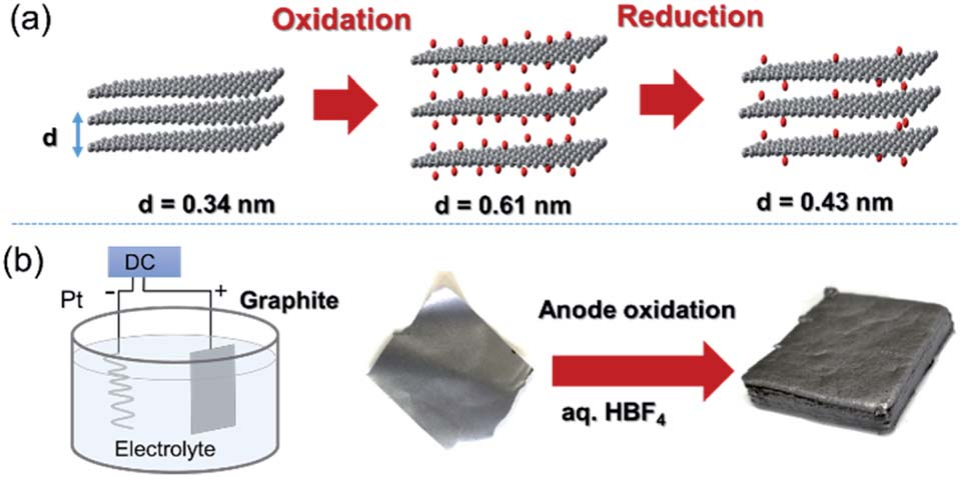
(a) Tuning the interlayer distance of graphene. (b) Production of a uniformly expanded graphene material by electrochemical treatment.

Graphite is composed of graphene sheets with a distance of 0.34 nm between each interlayer. This distance can be expanded by intercalation and chemical modification of the graphene layers. Chemical oxidation with a KMnO_4_/H_2_SO_4_ system and thermal reduction of GO are generally used to control the interlayer distance of graphene (Fig. 6a),^44^ while recent progress in the electrochemical redox system can provide a uniformly expanded graphene material (Fig. 6b).^45^ Such expanded graphene material is expected to be applied for electrodes in next-generation batteries, in which larger ions than Li are used.

**Fig. 7:**
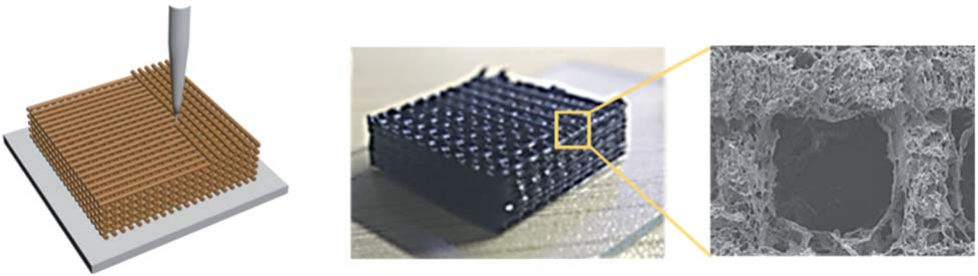
3D printing of graphene ink and the product. Reproduced with permission from ref. 47. Copyright 2018 Wiley-VCH Verlag GmbH & Co. KGaA.

3D printing technology has been adopted to construct 3D macro structures. By using graphene ink for a 3D printer, it was demonstrated that a free-standing graphene material could be constructed.^46^ Similarly, periodic deposition of GO inks produced periodic graphene aerogel gratings by 3D printing (Fig. 7).^47^ The key to the 3D printing process is the preparation of the ink. Now the preparation methods of graphene materials have almost been established, and the research phase is now shifting to determine more practical applications.

## 6. Electrocatalytic applications of carbon-rich materials with 3D ordering

Electrocatalysts are among the most attractive applications of 3D carbons owing to their excellent electron conductivity and large surface area. In this section, we discuss the unique electrocatalytic activity and selectivity of carbon-rich materials with 3D ordering.

**Fig. 8:**
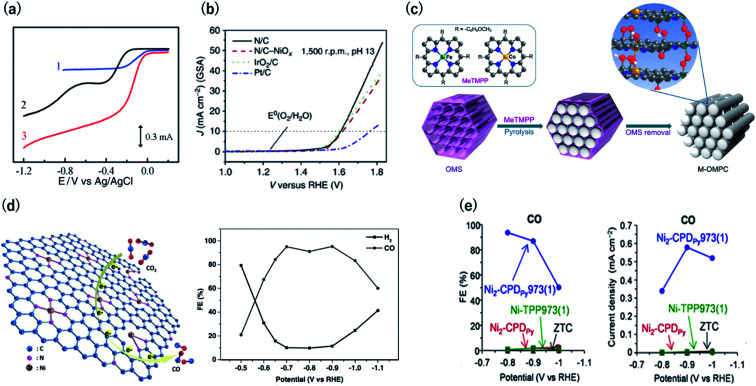
(a) Current *vs.* potential curves in 0.1 M KOH for the Pt/C (curve 1), nitrogen-free vertically aligned carbon nanotubes supported by a glassy carbon electrode (curve 2), and vertically aligned nitrogen-doped carbon nanotubes (curve 3). Reproduced with permission from ref. 50. Copyright 2009 AAAS. (b) Oxygen evolution activity for nitrogen-doped carbon in 0.1 M KOH, IrO_2_/C, and Pt/C. (c) Synthesis of ordered mesoporous porphyrinic carbons. Reproduced with permission from ref. 51 and 55. Copyright 2013 Nature Publishing Group, respectively. (d) (left) CO_2_ reduction reaction on Ni–N-graphene and (right) faradaic efficiency of CO generation by Ni–N-graphene in CO_2_-saturated 0.1 M KHCO_3_. Reproduced with permission from ref. 58. Copyright 2016 Wiley-VCH Verlag GmbH & Co. KGaA. (e) (left) Faradaic efficiency and (right) partial current density for CO by OCFs-600 in CO_2_-saturated 0.1 M KHCO_3_. Reproduced with permission from ref. 12. Copyright 2017 Nature Publishing Group.

Oxygen reduction and evolution (*i.e.*, water oxidation) are key reactions for the cathode of various fuel cells and the anode of artificial photosynthesis, respectively. Thus, it is important to discover efficient and cost-effective electrocatalysts for the oxygen reduction (ORR) and evolution reactions (OER) from the viewpoint of energy.^48^,^49^ Carbon-based ORR/OER electrocatalysts are divided into two categories: metal-free and 3d metal doped carbons. As for the metal-free materials, in 2009, it was revealed that nitrogen-doped carbons are efficient ORR electrocatalysts by using vertically aligned nitrogen-containing carbon nanotubes (CNTs) in alkaline electrolytes (Fig. 8a).^50^ Subsequently, Zhao *et al.* clearly demonstrated that N-doped carbon can also efficiently catalyze water oxidation catalysts in 2013.^51^,^52^ They synthesized N-doped graphite nanomaterials by the pyrolysis of a melamine/formaldehyde polymer. The N-doped carbons showed a current density of 10 mA cm^–2^ for OER at an overpotential of 0.38 V *vs.* RHE, which are values that are comparable to those of cobalt and iridium dioxides in 0.1 M KOH (Fig. 8b). Therefore, N–C can serve as bifunctional materials catalyzing both ORR and OER in alkaline solutions (Fig. 8a and b, respectively). In contrast to metal-free carbons, since the 1970s, it has been known that 3d metals (Fe and Co)-nitrogen co-doped carbons exhibit efficient ORR catalytic activity even in acidic solutions.^53^,^54^ For example, Jae *et al.* synthesized ordered mesoporous porphyrinic carbons containing Fe and Co by using nanocasting of mesoporous silica templates (Fig. 8c).^55^ The resulting Fe and Co co-doped carbons exhibited an efficient ORR activity in acidic solutions with an onset-potential of 0.9 V *vs.* RHE. They suggested that the bridging species (*i.e.*, M–(O_2_)–M) between the interlayers of the mesoporous structure facilitated the ORR.

The electrochemical carbon dioxide reduction reaction (CO_2_RR) in aqueous electrolytes is a promising technology using CO_2_ as an alternative carbon feedstock. Although Hori *et al.* reported that Cu metal electrodes selectively reduced CO_2_ to hydrocarbons in 1990s, such electrodes show a faradaic efficiency (FE) of over 20% for the competitive hydrogen evolution reactions.^56^ Recently, M–N–C based electrocatalysts have been reported to reduce CO_2_ to CO with high FE. Strasser *et al.* reported that metal (Fe, Mn)-containing N-doped porous carbon black-based solid catalysts show CO and methane production and proposed that N moieties serve as active sites for CO production.^57^ Su *et al.* were the first to synthesize single Ni atoms coordinated with N in carbon-based materials as a CO_2_RR catalyst.^58^ They doped the Ni–N bonds in 2D graphene nanosheets by the short-duration heat treatment of a Ni–organometallic complex to suppress the breakage of Ni–N coordination bonds of the precursors. The resulting catalyst showed efficient CO production with FE of over 90% at –0.7 to –0.9 V *vs.* RHE (Fig. 8d). This is because the short-duration heat treatment suppresses the formation of Ni nanoparticles with high HER activity. Based on this knowledge, we newly developed a method for the carbonization of organic crystal to form crystalline carbons (Fig. 3). Owing to the high content of resulting Ni–N_4_ sites in crystalline carbon, the FE for CO_2_RR reached up to 94% at –0.8 V *vs.* RHE (Fig. 8e).^12^ In contrast, the conventional Ni containing N–C catalysts synthesized by the pyrolysis of common amorphous porphyrin, Ni-tetraphenylporphyrin (TPP) exhibited a poor CRR activity (FE < 10%) because TPP was decomposed to form the Ni nanoparticle with a high HER activity during the heat treatment. The conventional M–N–C catalysts have an ambiguous structure, and even the active sites for ORR and CO_2_RR are unclear. However, crystalline carbons derived from organic crystal have a defined structure, and thus, they are desirable electrocatalysts not only for the high activity but for clarifying the active center, which provides us with a sophisticated design strategy.

## 7. Summary and outlook

In this mini-review, we describe the preparation of carbon-rich materials with 3D ordering at the angstrom level. Porous carbons that retain the original cavity size of pillar[6]arene at the angstrom level were successfully synthesized by calcination of a hexagonal assembly of pillar[6]arenes. A thermally stable hexagonal close-packed structure is the key to retain the pillar[6]arene cavity even after calcination. Calcination of cyclic porphyrin dimer assemblies afforded crystalline carbons because of the high thermal stability of porphyrin units and thermal polymerization of diacetylene groups. These new pathways are a starting point for designing carbon-rich materials with 3D ordering by calcination of supramolecular assemblies based on a rational molecular design. Preparation of carbon-rich materials from designed monomers is a bottom-up approach, and is useful to prepare carbon-rich materials containing heteroatoms, such as nitrogen atoms, at a desired position. The electrochemical redox reaction of graphene is a top-down approach to create carbon-rich materials with control at the angstrom level, and should be a powerful method for their mass production. An electrocatalytic reaction using such carbon-rich materials is a useful application, and they will also be potentially applied as adsorbents, catalyst supports, and electrode materials.

## Conflicts of interest

There are no conflicts to declare.

## References

[cit1] Wu D., Xu F., Sun B., Fu R., He H., Matyjaszewski K. (2012). Chem. Rev..

[cit2] Wagle D. V., Zhao H., Baker G. A. (2014). Acc. Chem. Res..

[cit3] Nishihara H., Kyotani T. (2012). Adv. Mater..

[cit4] Titirici M.-M., Antonietti M. (2010). Chem. Soc. Rev..

[cit5] Lee J., Kim J., Hyeon T. (2006). Adv. Mater..

[cit6] Nishihara H., Yang Q.-H., Hou P.-X., Unno M., Yamauchi S., Saito R., Paredes J. I., Martínez-Alonso A., Tascón J. M. D., Sato Y., Terauchi M., Kyotani T. (2009). Carbon.

[cit7] Paraknowitsch J. P., Zhang J., Su D., Thomas A., Antonietti M. (2010). Adv. Mater..

[cit8] Radhakrishnan L., Reboul J., Furukawa S., Srinivasu P., Kitagawa S., Yamauchi Y. (2011). Chem. Mater..

[cit9] Zhang W., Wu Z.-Y., Jiang H.-L., Yu S.-H. (2014). J. Am. Chem. Soc..

[cit10] Zou F., Hu X., Li Z., Qie L., Hu C., Zeng R., Jiang Y., Huang Y. (2014). Adv. Mater..

[cit11] Ogoshi T., Yoshikoshi K., Sueto R., Nishihara H., Yamagishi T. (2015). Angew. Chem., Int. Ed..

[cit12] Nishihara H., Hirota T., Matsuura K., Ohwada M., Hoshino N., Akutagawa T., Higuchi T., Jinnai H., Koseki Y., Kasai H., Matsuo Y., Maruyama J., Hayasaka Y., Konaka H., Yamada Y., Yamaguchi S., Kamiya K., Kamimura T., Nobukuni H., Tani F. (2017). Nat. Commun..

[cit13] Sakamoto R., Fukui N., Maeda H., Matsuoka R., Toyoda R., Nishihara H. (2019). Adv. Mater..

[cit14] Sakamoto R., Shiotsuki R., Wada K., Fukui N., Maeda H., Komeda J., Sekine R., Harano K., Nishihara H. (2018). J. Mater. Chem. A.

[cit15] Wang Z., Gao H., Zhang Q., Liu Y., Chen J., Guo Z. (2019). Small.

[cit16] Tan L., Tan B. (2017). Chem. Soc. Rev..

[cit17] Wang X., Mu P., Zhang C., Chen Y., Zeng J., Wang F., Jiang J.-X. (2017). ACS Appl. Mater. Inter..

[cit18] Luo Y., Li B., Wang W., Wu K., Tan B. (2012). Adv. Mater..

[cit19] Lee J.-S. M., Briggs M. E., Hasell T., Cooper A. I. (2016). Adv. Mater..

[cit20] Zhang C., Kong R., Wang X., Xu Y., Wang F., Ren W., Wang Y., Su F., Jiang J.-X. (2017). Carbon.

[cit21] Zhang Q.-M., Zhai T.-L., Wang Z., Cheng G., Ma H., Zhang Q.-P., Zhao Y.-H., Tan B., Zhang C. (2019). Adv. Mater. Inter..

[cit22] Zhai T.-L., Tan L., Luo Y., Liu J.-M., Tan B., Yang X.-L., Xu H.-B., Zhang C. (2016). Chem.–Asian J..

[cit23] Nobukuni H., Shimazaki Y., Tani F., Naruta Y. (2007). Angew. Chem., Int. Ed..

[cit24] Nishihara H., Matsuura K., Ohwada M., Yamamoto M., Matsuo Y., Maruyama J., Hayasaka Y., Yamaguchi S., Kamiya K., Konaka H., Inoue M., Tani F. (2020). Chem. Lett..

[cit25] Narita A., Wang X.-Y., Feng X., Müllen K. (2015). Chem. Soc. Rev..

[cit26] Segawa Y., Ito H., Itami K. (2016). Nat. Rev. Mater..

[cit27] Novoselov K. S., Geim A. K., Morozov S. V., Jiang D., Zhang Y., Dubonos S. V., Grigorieva I. V., Firsov A. A. (2004). Science.

[cit28] Jia Z., Li Y., Zuo Z., Liu H., Huang C., Li Y. (2017). Acc. Chem. Res..

[cit29] Gao X., Liu H., Wang D., Zhang J. (2019). Chem. Soc. Rev..

[cit30] Li G., Li Y., Liu H., Guo Y., Li Y., Zhu D. (2010). Chem. Commun..

[cit31] Matsuoka R., Sakamoto R., Hoshiko K., Sasaki S., Masunaga H., Nagashio K., Nishihara H. (2017). J. Am. Chem. Soc..

[cit32] Sakamoto R., Hoshiko K., Liu Q., Yagi T., Nagayama T., Kusaka S., Tsuchiya M., Kitagawa Y., Wong W.-Y., Nishihara H. (2015). Nat. Commun..

[cit33] Kambe T., Sakamoto R., Hoshiko K., Takada K., Miyachi M., Ryu J.-H., Sasaki S., Kim J., Nakazato K., Takata M., Nishihara H. (2013). J. Am. Chem. Soc..

[cit34] Sun X., Wu K.-H., Sakamoto R., Kusamoto T., Maeda H., Ni X., Jiang W., Liu F., Sasaki S., Masunaga H., Nishihara H. (2017). Chem. Sci..

[cit35] Pal T., Doi S., Maeda H., Wada K., Tan C. M., Fukui N., Sakamoto R., Tsuneyuki S., Sasaki S., Nishihara H. (2019). Chem. Sci..

[cit36] Zhao Y., Wan J., Yao H., Zhang L., Lin K., Wang L., Yang N., Liu D., Song L., Zhu J., Gu L., Liu L., Zhao H., Li Y., Wang D. (2018). Nat. Chem..

[cit37] Liu R., Liu H., Li Y., Yi Y., Shang X., Zhang S., Yu X., Zhang S., Cao H., Zhang G. (2014). Nanoscale.

[cit38] He J., Wang N., Cui Z., Du H., Fu L., Huang C., Yang Z., Shen X., Yi Y., Tu Z., Li Y. (2017). Nat. Commun..

[cit39] Cao X., Yin Z., Zhang H. (2014). Energy Environ. Sci..

[cit40] Gorgolis G., Galiotis C. (2017). 2D Mater..

[cit41] Li C., Shi G. (2012). Nanoscale.

[cit42] Lian B., Deng J., Leslie G., Bustamante H., Sahajwalla V., Nishina Y., Joshi R. K. (2017). Carbon.

[cit43] Saito A., Yamamoto S., Nishina Y. (2014). RSC Adv..

[cit44] Wen Y., He K., Zhu Y., Han F., Xu Y., Matsuda I., Ishii Y., Cumings J., Wang C. (2014). Nat. Commun..

[cit45] Campéon B. D. L., Akada M., Ahmad M. S., Nishikawa Y., Gotoh K., Nishina Y. (2020). Carbon.

[cit46] Kim J. H., Chang W. S., Kim D., Yang J. R., Han J. T., Lee G.-W., Kim J. T., Seol S. K. (2015). Adv. Mater..

[cit47] Jiang Y., Xu Z., Huang T., Liu Y., Guo F., Xi J., Gao W., Gao C. (2018). Adv. Funct. Mater..

[cit48] Roger I., Shipman M. A., Symes M. D. (2017). Nat. Rev. Chem..

[cit49] Zhu C., Li H., Fu S., Du D., Lin Y. (2016). Chem. Soc. Rev..

[cit50] Gong K., Du F., Xia Z., Durstock M., Dai L. (2009). Science.

[cit51] Zhao Y., Nakamura R., Kamiya K., Nakanishi S., Hashimoto K. (2013). Nat. Commun..

[cit52] Zhao Y., Kamiya K., Hashimoto K., Nakanishi S. (2015). J. Phys. Chem. C.

[cit53] Jahnke H., Schonborn M., Zimmermann G. (1976). Top. Curr. Chem..

[cit54] Kamiya K., Hashimoto K., Nakanishi S. (2012). Chem. Commun..

[cit55] Cheon J. Y., Kim T., Choi Y., Jeong H. Y., Kim M. G., Sa Y. J., Kim J., Lee Z., Yang T.-H., Kwon K., Terasaki O., Park G.-G., Adzic R. R., Joo S. H. (2013). Sci. Rep..

[cit56] Hori Y., Wakebe H., Tsukamoto T., Koga O. (1994). Electrochim. Acta.

[cit57] Varela A. S., Ranjbar Sahraie N., Steinberg J., Ju W., Oh H.-S., Strasser P. (2015). Angew. Chem., Int. Ed..

[cit58] Su P., Iwase K., Nakanishi S., Hashimoto K., Kamiya K. (2016). Small.

